# Genome Sequences of Antigenically Distinct Serotype O Foot-and-Mouth Disease Viruses from Pakistan

**DOI:** 10.1128/MRA.01397-18

**Published:** 2019-01-17

**Authors:** Katarzyna Bachanek-Bankowska, Jemma Wadsworth, Elisabeth Henry, Anna B. Ludi, Abdelghani Bin-Tarif, Bob Statham, Donald P. King, Muhammad Afzal, Manzoor Hussain, Shumaila Manzoor, Muhammad Abubakar, Nick J. Knowles

**Affiliations:** a The Pirbright Institute, Surrey, United Kingdom; b Food and Agriculture Organization of the United Nations, Pakistan Office, Islamabad, Pakistan; c National Veterinary Laboratories, Islamabad, Pakistan; University of Maryland School of Medicine

## Abstract

The genome sequences of three serotype O foot-and-mouth disease viruses (FMDVs) isolated from outbreaks in Pakistan in 2016 and 2017 are described. Despite all three isolates being classified in the same FMDV genetic sublineage, two of them displayed a distinct antigenic phenotype against commonly used vaccine strains.

## ANNOUNCEMENT

Foot-and-mouth disease (FMD) virus (FMDV) is a single-stranded positive-sense RNA virus of the genus Aphthovirus (family Picornaviridae), the etiological agent of a highly contagious disease of livestock affecting animal production and trade throughout Asia and Africa. Vaccination with an antigenically matched vaccine is one of the most important interventions for FMD prevention and control.

In September 2017, the FAO World Reference Laboratory for FMD (WRLFMD) received samples collected in Pakistan, including the following three specimens collected in the Punjab region: PAK/10/2016 from cattle sampled on 5 December 2016, PAK/4/2017 from water buffalo sampled on 2 February 2017, and PAK/14/2017 from cattle sampled on 3 November 2017. FMDV type O was isolated from each sample using primary bovine thyroid cells (1). Phylogenetic analysis based on the VP1 coding sequences of all three viruses classified them within the Middle East-South Asia (ME-SA) topotype, PanAsia-2 lineage, and ANT-10 sublineage (O/ME-SA/PanAsia-2^ANT-10^) (2). Viruses of this sublineage have been previously reported in Afghanistan, Bulgaria, Israel, Iran, Pakistan, Saudi Arabia, and Turkey.

Antigenic matching, assessed by virus neutralization, is expressed as an “r1” value, a relationship coefficient of antibody titers obtained using sera from vaccinated animals against both the vaccine strain and a FMDV field isolate (3). Vaccine matching of the three virus isolates was performed using antisera raised against three vaccines strains commonly used in the affected area (O 3039 and O Manisa [Boehringer Ingelheim] and O TUR 5/09 [MSD]). The vaccine-matching results for O/PAK/14/2017 were indicative of an antigenic match for all the vaccines (r1 values between 0.32 and 0.62, greater than the threshold of 0.3), while those of the other two isolates were almost nonreactive in the variable virus neutralization test (VNT) (r1 values between 0 and 0.1).

Here, we report the complete genome sequences of the three isolates obtained using MiSeq technology (Illumina, USA) as previously described (4). A paired-end next-generation sequencing (NGS) run of 2 × 150-nucleotide (nt) read length were obtained, and sequences were assembled using SeqMan NGen and SeqMan Pro using default settings (Lasergene package version 15; DNAStar, Inc., Madison, WI). *De novo* assembly of 0.67, 0.53, and 0.6 million total generated reads resulted in 88,000, 48,000, and 63,000 reads that formed the three genome sequences of O/PAK/10/2016 (8,193 nt; G+C content, 53.8%), O/PAK/4/2017 (8,194 nt; G+C content, 53.9%), and O/PAK/14/2017 (8,197 nt; G+C content, 53.7%), respectively. These genomes included 15 nt of a poly(C) tract of unknown length located within the 5′ untranslated region (UTR) and 10 nt of a poly(A) tail at the 3′ end of the genome. A single, large open reading frame of 6,999 nt was predicted to encode a polyprotein of 2,333 amino acids (aa) containing 4 structural (VP4, VP2, VP3, and VP1) and 10 nonstructural (L, 2 A, 2B, 2 C, 3 A, 3B1, 3B2, 3B3, 3 C, and 3D) proteins. Genomes of both antigenically distinct viruses, O/PAK/10/2016 and O/PAK/4/2017, were closely related to each other, with 99.2% nt identity, and more distantly related to the O/PAK/14/2017 sequence, with 96.9% and 97.1% nt identity, respectively. Amino acid substitutions within the capsid at positions VP2^72^ (threonine to alanine) and VP2^132^ (isoleucine to methionine) were consistent with the involvement of antigenic site 2 (5) and may reflect the low r1 values observed. The emergence of what appears to be a new antigenic variant of serotype O was unexpected. The spread of antigenically similar viruses needs to be closely monitored in order to understand any potential impacts upon FMDV vaccination campaigns in the region.

### Data availability.

The nucleotide sequences of FMDV O/PAK/10/2016, O/PAK/4/2017, and O/PAK/14/2017 were deposited in GenBank under accession no. MH784403, MH784404, and MH784405, respectively. The raw sequence data were deposited in the NCBI Sequence Read Archive under BioProject accession no. PRJNA506001.
